# Reciprocal effects of capsaicin and menthol on thermosensation through regulated activities of TRPV1 and TRPM8

**DOI:** 10.1007/s12576-015-0427-y

**Published:** 2015-12-08

**Authors:** Masayuki Takaishi, Kunitoshi Uchida, Yoshiro Suzuki, Hiroshi Matsui, Tadashi Shimada, Fumitaka Fujita, Makoto Tominaga

**Affiliations:** Division of Cell Signaling, Okazaki Institute for Integrative Bioscience, National Institutes of Natural Sciences, Higashiyama 5-1, Myodaiji, Okazaki, Aichi 444-8787 Japan; Mandom Corporation, Osaka, 540-8530 Japan; Department of Physiological Sciences, The Graduate University for Advanced Studies (SOKENDAI), Okazaki, 444-8585 Japan

**Keywords:** Menthol, Capsaicin, TRPV1, TRPM8, Analgesic

## Abstract

Transient receptor potential vanilloid 1 (TRPV1) is activated by elevated temperature (>42 °C), and it has been reported that cold temperature decreases capsaicin-induced TRPV1 activity. In contrast, transient receptor potential melastatin 8 (TRPM8) is activated by low temperatures and menthol, and heat stimulation suppresses menthol-evoked TRPM8 currents. These findings suggest that the effects of specific agents on TRPV1 and TRPM8 channels are intricately interrelated. We examined the effects of menthol on human (h)TRPV1 and of capsaicin on hTRPM8. hTRPV1 currents activated by heat and capsaicin were inhibited by menthol, whereas hTRPM8 currents activated by cold and menthol were similarly inhibited by capsaicin. An in vivo sensory irritation test showed that menthol conferred an analgesic effect on the sensory irritation evoked by a capsaicin analogue. These results indicate that in our study the agonists of TRPV1 and TRPM8 interacted with both of these channels and suggest that the anti-nociceptive effects of menthol can be partially explained by this phenomenon.

**Electronic supplementary material:**

The online version of this article (doi:10.1007/s12576-015-0427-y) contains supplementary material, which is available to authorized users.

## Introduction

Transient receptor potential (TRP) channels respond to a wide variety of sensory stimuli, including temperature, nociceptive compounds, touch, osmolarity, and pheromones [1–3]. In particular, the involvement of TRP channels in thermosensation became the subject of extensive study after the cloning of the receptor of capsaicin [transient receptor potential vanilloid type 1 (TRPV1)], a pungent component of hot chili peppers [4, 5]. TRPV1 is activated by exogenous agonists (capsaicin and resiniferatoxin) and by physical stimuli, such as heat (>42 °C) [6]. Transient receptor potential melastatin 8 (TRPM8), on the other hand, has been proposed to be a sensor of low temperatures in the innocuous to noxious ranges [7–10]. This channel is also activated by various cooling agents, such as menthol and icilin [7, 11, 12], by voltage [13, 14], and by phosphatidylinositol 4,5-bisphosphate (PIP_2_) which regulates the activity of a variety of ion channels [15–18]. Approximately 10 % of small-diameter sensory neurons in the rat express TRPM8 but not TRPV1 [7, 19], which is consistent with observations of no TRPM8 and TRPV1 colocalization in the majority of studies using TRPM8 antibodies [20–23]. These results suggest that different nerve fibers are devoted to sensing cold and hot temperatures [24].

It has been reported that low temperatures decrease capsaicin-induced TRPV1 activation [25], whereas heat stimulation suppresses menthol-evoked current via TRPM8 [26], indicating that thermal stimulation has opposite effects on TRPV1 and TRPM8. These opposite effects suggest that input of thermal information may be regulated in a complementary manner through changes in thermosensitive TRP channel activities. As such, this notion suggests mechanisms for reciprocal effects on TRPM8 and TRPV1 in sensory systems.

Capsaicin causes heat sensation through TRPV1 activation, and menthol produces a cool sensation through TRPM8 activation; therefore, these two chemicals elicit opposite thermosensation reactions. Several other chemicals also exhibit opposing effects on the two channels; for example, ethanol [27], spermine [15, 28], and acid [6, 29, 30] inhibit TRPM8, whereas they potentiate the activity of TRPV1. Furthermore, PIP_2_ inhibits TRPV1 at high concentrations, whereas it activates TRPM8 [31]. These data suggest that the effects of some chemicals on the TRPV1 and TRPM8 channels intricately interact with each other. Therefore, we hypothesized that menthol and capsaicin also have inhibitory effects on TRPV1 and TRPM8, respectively, in a manner similar to thermal stimulation.

Menthol has been used for its anti-nociceptive effects for more than one thousand years [32], and preparations containing menthol are used topically to relieve neuralgia in traditional Chinese and European medicine [33]. Menthol also demonstrates some anesthetic [34–36] and κ-opioid-mediated anti-nociceptive properties in mouse hot-plate tests [37]. Moreover, it has been shown that TRPM8 contributes to mediation of the effects of cold analgesia in the rat [38] and anti-nociception in mice [39]. However, the anti-nociceptive mechanisms of menthol are not yet fully understood. Since TRPV1 acts as an integrator of painful stimuli, TRPV1 antagonists can be considered as promising novel types of analgesics in therapeutic regimen [40–43].

In this study, we examined the effects of menthol on human TRPV1 (hTRPV1) and the effects of capsaicin on hTRPM8. The hTRPV1-mediated currents induced by capsaicin were inhibited by menthol in a dose-dependent manner, whereas the hTRPM8-mediated currents induced by menthol were inhibited by capsaicin in a dose-dependent manner. The results of an in vivo sensory irritation test showed that menthol conferred an analgesic effect on the sensory irritation produced by vanillyl butyl ether (VBE), a capsaicin analogue. Based on these results, we propose a new concept, namely, that capsaicin and menthol exhibit mutually opposing effects on these channels.

## Materials and methods

### Molecular cloning

Full-length *hTRPV1* and *hTRPM8* were obtained from Life Technologies Corp. (Carlsbad, CA). cDNAs were cloned into the pcDNA3.1 vector.

### Cell culture

Human embryonic kidney (HEK) 293T cells were maintained in Dulbecco's Modified Eagle medium (DMEM; Wako Pure Chemical Industries Ltd., Osaka, Japan) supplemented with 10 % fetal bovine serum (FBS; Biowest SAS, Caille, France), 100 U/mL penicillin (Life Technologies Corp.), 100 µg/mL streptomycin (Life Technologies Corp.), and 2 mM l-glutamine (GlutaMAX; Life Technologies Corp.), at 37 °C in 5 % CO_2_. For Ca^2+^-imaging, 1 µg of plasmid DNA containing hTRPV1 and hTRPM8 in pcDNA3 in OPTI-MEM medium (Life Technologies Corp.) was transfected into HEK293T cells using Lipofectamine Plus Reagent (Life Technologies Corp.). After a 3- to 4-h incubation, cells were reseeded on coverslips and were incubated further at 37 °C in 5 % CO_2_.

### hTRPV1 mutants and hTRPM8 mutants

Three types of hTRPV1 mutants (Y511A, S512Y, T550I) were constructed using a modified QuickChange Site-Directed Mutagenesis method (Agilent Technologies Inc., Santa Clara, CA) [44–46], and three types of hTRPM8 mutants (Y745H, Y1005F, and L1009R) were constructed by single amino acid substitutions using a GeneTailor Site-Directed Mutagenesis System (Invitrogen) [27, 47, 48]. The entire sequences, including the desired substitutions in the mutants, were confirmed.

### Calcium ion-imaging

Calcium ion (Ca^2+^)-imaging was performed 1 day after transfection. HEK293T cells on coverslips were mounted in an open chamber and superfused with a standard bath solution (140 mM NaCl, 5 mM KCl, 2 mM MgCl_2_, 2 mM CaCl_2_, 10 mM HEPES, and 10 mM glucose, pH 7.4). Cytosolic-free Ca^2+^ concentrations in HEK293T cells were measured by dual-wavelength microfluorometry using the Fura-2 radiometric indicator (Molecular Probes, Invitrogen Corp.) with excitation at 340/380 nm and emission at 510 nm. The Fura-2 ratio image was calculated and acquired using the IP-Lab Imaging Processing system (Scanalytics Inc., Fairfax, VA, USA).

### Electrophysiology

Whole-cell patch-clamp recordings were performed 1 day after transfection. The standard bath solution was the same as that used in the Ca^2+^-imaging experiments; extracellular Ca^2+^ was removed and 5 mM EGTA was added for experiments in which our aim was to determine the dose-dependent effects of menthol or capsaicin. The pipetted solution contained 140 mM KCl, 5 mM EGTA, and 10 mM HEPES, pH 7.4 (adjusted with KOH). Data from the whole-cell voltage-clamp recordings were sampled at 10 kHz and filtered at 5 kHz for analysis (Axon 200B amplifier with pCLAMP software; Axon Instruments, Sunnyvale, CA). The membrane potential was clamped at −60 mV for all conditions. Voltage ramp-pulses from −150 to +100 mV (500 ms) were applied every 5 s for the inhibition of TRPV1 or TRPM8 activated by capsaicin or menthol, respectively, and every 2.5 s for the inhibition of TRPV1 or TRPM8 activated by temperatures or VBE, respectively.

### Human subjects

Japanese male subjects 20–30 years old were selected as participants to eliminate confounding factors that may influence the perception of sensitive skin, including race, age, gender, and hormonal and psychosocial interactions. To evaluate sensory irritation, we first randomly selected 10 of the 22 skin-sensitive male volunteers—from among a total of 49 male subjects examined who were able to discriminate the effects of menthol on the skin at between 0.05 and 0.1 wt% menthol. Female volunteers were excluded because of possible hormonal interactions. Informed consent was obtained from all participants, and the study was approved by the Ethical Committee of Mandom Corp.

### Sensory irritation tests

The study was conducted at a temperature of 21–23 °C and a relative humidity of 45–55 %. The side of the neck, which is innervated by spinal nerves, was the target of the skin irritation analysis because this region is known to be sensitive to various skin irritants [49]. Prior to testing, areas of skin were cleaned with a wet towel and acclimatized for 10. Blind randomized half-region (left vs. right) trials were performed with two different samples applied to the neck region. A total of 80 µL of base was applied. The subjects evaluated pricking, stinging, burning, and itching sensations after 1, 3, 5, 7, 9, and 10 min of compound/chemical application in accordance with the criteria summarized in Table 1. The total sensory irritation scores were calculated for the entire period.

**Table 1:**
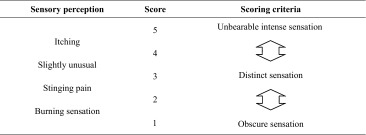
Sensory irritation scores

### Data analysis

Data in all of the figures are shown as the mean ± standard error of the mean (SEM), and *p* values < of 0.05 were considered significant. Statistical significance of effects of menthol and capsaicin on hTRPV1 and hTRPM8 mutants were evaluated using Student’s *t* test. Dose-dependent curves were fit with a Hill equation. Sensory irritation tests were evaluated using a Wilcoxon signed-rank test.

## Results

### Menthol inhibited hTRPV1 activity induced by capsaicin, and capsaicin inhibited hTRPM8 activity induced by menthol

**Fig. 1:**
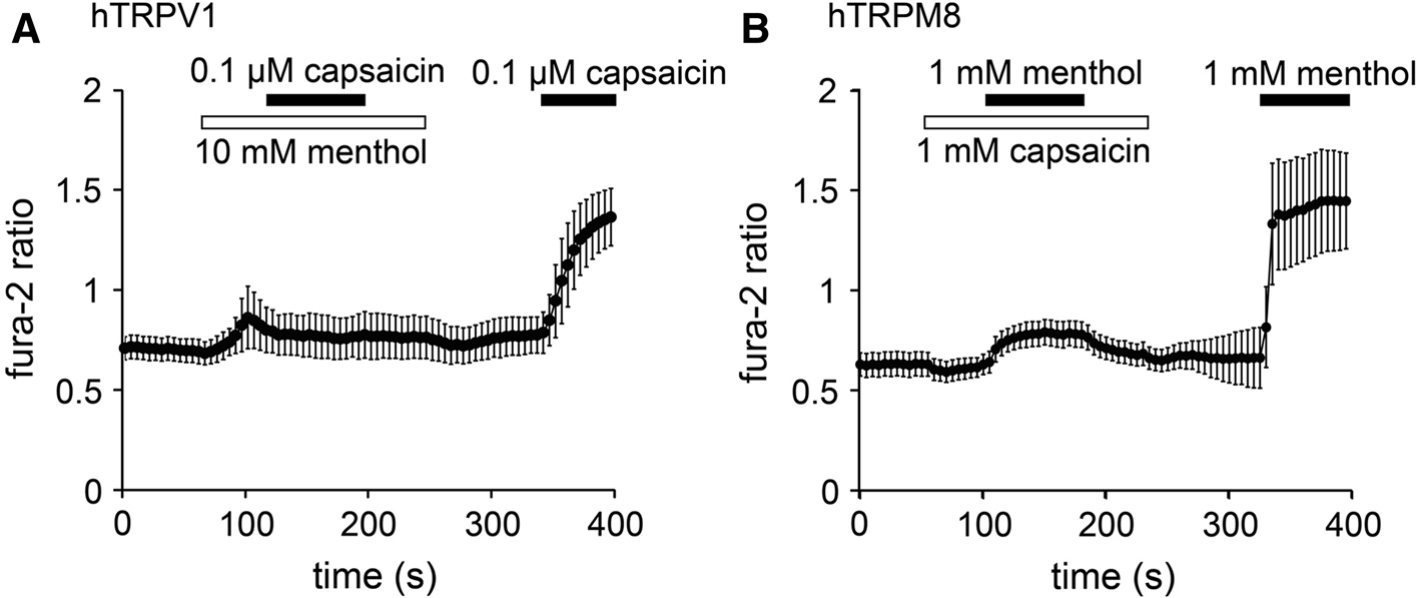
Effects of menthol and capsaicin on human transient receptor potential vanilloid 1 (*hTRPV1*) or human transient receptor potential melastatin* (hTRPM8*). **a** Changes in the Fura-2 ratio in response to capsaicin (0.1 µM) application in the presence and absence of menthol (10 mM) in human embryonic kidney (HEK) 293T cells expressing hTRPV1 (*n* = 40). **b** Changes in Fura-2 ratio in response to menthol (1 mM) application in the presence and absence of capsaicin (1 mM) in HEK293T cells expressing hTRPM8 (*n* = 94). Data are shown as the mean ± standard deviation (SD)

We first examined the effects of menthol on hTRPV1 and the influence of capsaicin on hTRPM8 in HEK-derived 293T (HEK293T) cells expressing hTRPV1 or hTRPM8 using a Ca^2+^-imaging method at room temperature. In cells expressing hTRPV1, the changes in the Fura-2 ratios (i.e., cytosolic Ca^2+^ concentrations [Ca^2+^]_i_) induced by capsaicin (0.1 µM) in the presence of menthol (10 mM) were smaller than those induced in the absence of menthol (Fig. 1a). Although there was a slight increase in [Ca^2+^]_i_ upon the application of menthol (10 mM) using a patch-clamp method we did not observe any current activation induced by 10 mM menthol in the cells expressing hTRPV1 nor in the vector-transfected cells [Electronic Supplementary Material (ESM) Fig. 1a, b], indicating that the slight [Ca^2+^]_i_ increase could be a non-specific phenomenon. In comparison, in cells expressing hTRPM8, changes in the Fura-2 ratios induced by menthol (1 mM) in the presence of capsaicin (1 mM) were smaller than those in the absence of capsaicin (Fig. 1b). Fura-2 ratios were slightly reduced by capsaicin alone in the cells expressing hTRPM8, likely because hTRPM8 was activated even at room temperature. We also confirmed that capsaicin (1 mM) did not cause current activation in the cells expressing hTRPM8 (ESM Fig. 1c). These results suggest that menthol inhibits hTRPV1 activity, whereas capsaicin inhibits hTRPM8 activity.

**Fig. 2:**
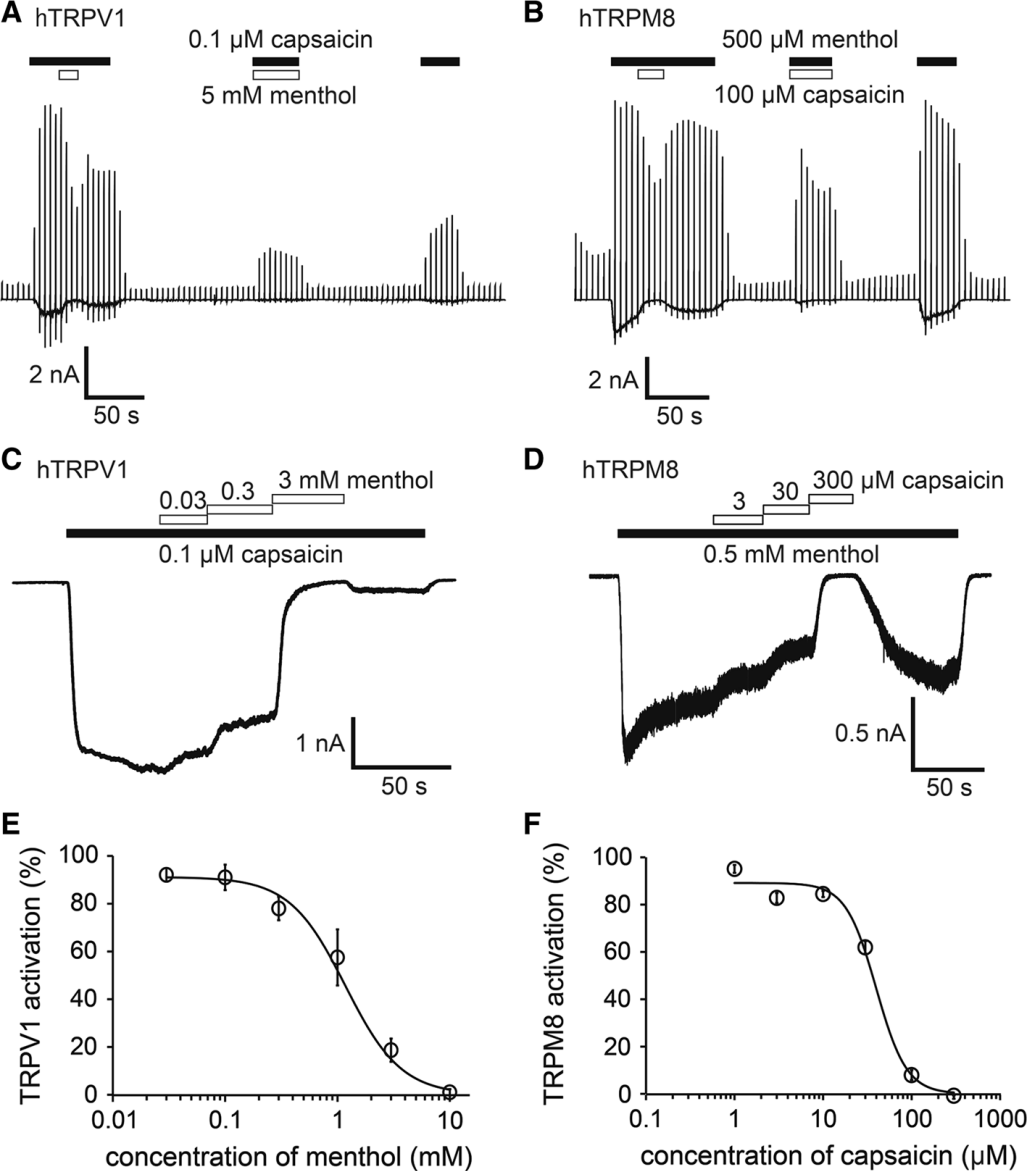
Inhibition of hTRPV1-mediated currents by menthol and inhibition of hTRPM8-mediated currents by capsaicin in HEK293T cells. **a** A representative trace of the whole cell showing that 0.1 µM capsaicin-evoked hTRPV1 currents were inhibited by menthol (5 mM) in the presence of extracellular Ca^2+^. **b** A representative trace of the whole cell showing that 500 µM menthol-evoked hTRPM8 currents were inhibited by 100 µM capsaicin in the presence of extracellular Ca^2+^. **c** A representative 0.1 μM capsaicin-evoked hTRPV1 current that was inhibited by menthol in a dose-dependent manner in the absence of extracellular Ca^2+^. **d** A representative 0.5 mM menthol-evoked hTRPM8 current that was inhibited by capsaicin in a dose-dependent manner in the absence of extracellular Ca^2+^. **e** Dose-dependent inhibition of 0.1 μM capsaicin-evoked hTRPV1 current by menthol. the half-maximal inhibitory concentration (IC_50_) and Hill’s coefficient values are 1.2 ± 0.2 mM and 1.7 ± 0.3, respectively (*n* = 5–8). **f** Dose-dependent inhibition of 0.5 mM menthol-evoked hTRPM8 current by capsaicin; the IC_50_ and Hill’s coefficient values are 39.9 ± 6.4 µM and 2.5 ± 0.7, respectively (*n* = 6–8). Data are shown as the mean ± standard error of the mean (SEM)

To confirm these possibilities, we performed patch–clamp experiments using HEK293T cells expressing hTRPV1 or hTRPM8. Capsaicin (0.1 µM) induced TRPV1-mediated current activation with an outwardly rectifying current–voltage (I–V) relationship revealed by ramp pulses from −150 to +100 mV every 5 s. The addition of menthol (5 mM) to the system reduced the capsaicin-evoked currents at both positive and negative potentials, and these currents were partly recovered by menthol washout (Fig. 2a). The currents evoked by concomitant application of capsaicin with menthol were smaller than those activated by capsaicin alone. The hTRPM8 currents induced by menthol (500 µM) were similarly inhibited by capsaicin (100 µM) in a reversible manner, and the currents evoked by concomitant application of menthol with capsaicin were smaller than those activated by menthol alone (Fig. 2b). These results are consistent with the data produced in the Ca^2+^-imaging experiments (Fig. 1).

We next examined the dose-dependency of the inhibitory effects. The current responses were measured in the absence of extracellular Ca^2+^ to minimize desensitization. Menthol inhibited 0.1 µM capsaicin-activated hTRPV1 currents in a dose-dependent manner, with a half-maximal inhibitory concentration (IC_50_) of approximately 1.2 ± 0.2 mM (Fig. 2c, e). The capsaicin-induced inhibition of menthol-activated hTRPM8 currents also occurred in a dose-dependent manner, with an IC_50_ of approximately 39.9 ± 6.4 µM (Fig. 2d, f). We also examined the effects of different concentrations of capsaicin on hTRPV1 in the absence and presence of 1 mM menthol and observed an apparent shift of the capsaicin dose-dependency towards high concentrations with menthol (ESM Fig. 2). These results indicate that menthol inhibited hTRPV1 activity and capsaicin inhibited hTRPM8 activity.

### Menthol and capsaicin inhibited the activation of hTRPV1 or hTRPM8 induced by thermal stimulation

**Fig. 3:**
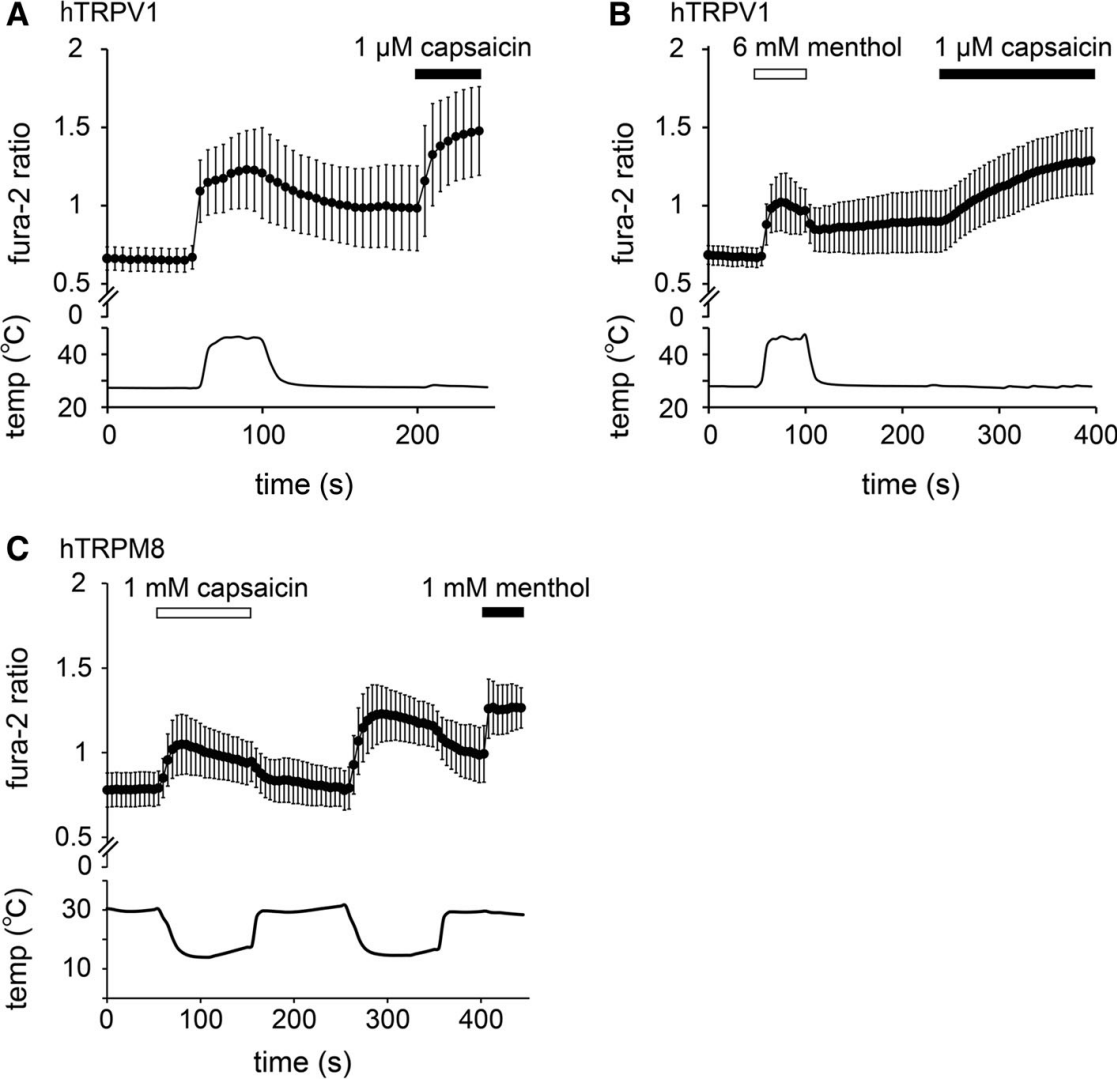
Inhibition of heat-evoked hTRPV1 and cold-evoked hTRPM8 responses by menthol and capsaicin. **a, b** Changes in the Fura-2 ratio in response to heat stimulation (>45 °C) in the absence (**a**) and presence (**b**) of menthol (6 mM) in HEK293T cells expressing hTRPV1 (*n* = 61). **c** Changes in the Fura-2 ratio in response to cold stimulation (<20 °C) in the presence and absence of capsaicin (1 mM) in HEK293T cells expressing hTRPM8 (*n* = 117). Data are shown as the mean ± SD

The inhibitory effects of menthol and capsaicin on hTRPV1 and hTRPM8 activities, respectively, induced by thermal stimulation were measured using a Ca^2+^-imaging method. To avoid the effects of desensitization during our investigation of the effects of menthol on heat-evoked (>45 °C) TRPV1-mediated [Ca^2+^]_i_ changes, we compared the effects in different cells. The increases in Fura-2 ratios induced by heat stimulation in the absence of menthol (0.54 ± 0.03) were significantly larger than those induced by heat stimulation in the presence of menthol (0.35 ± 0.02; *p* < 0.01) (Fig. 3a, b), suggesting that menthol inhibited hTRPV1 activity induced by heat stimulation. The increases in Fura-2 ratios induced by capsaicin after heat stimulation in the presence of menthol were slow, possibly because the effects of menthol were not completely abolished by washout. Similarly, in cells expressing hTRPM8, [Ca^2+^]_i_ increases caused by cold stimulation (<20 °C) in the presence of capsaicin (1 mM) were significantly smaller than those in the absence of capsaicin (0.19 ± 0.01 vs. 0.44 ± 0.01, respectively; *p* < 0.01) (Fig. 3c).

**Fig. 4:**
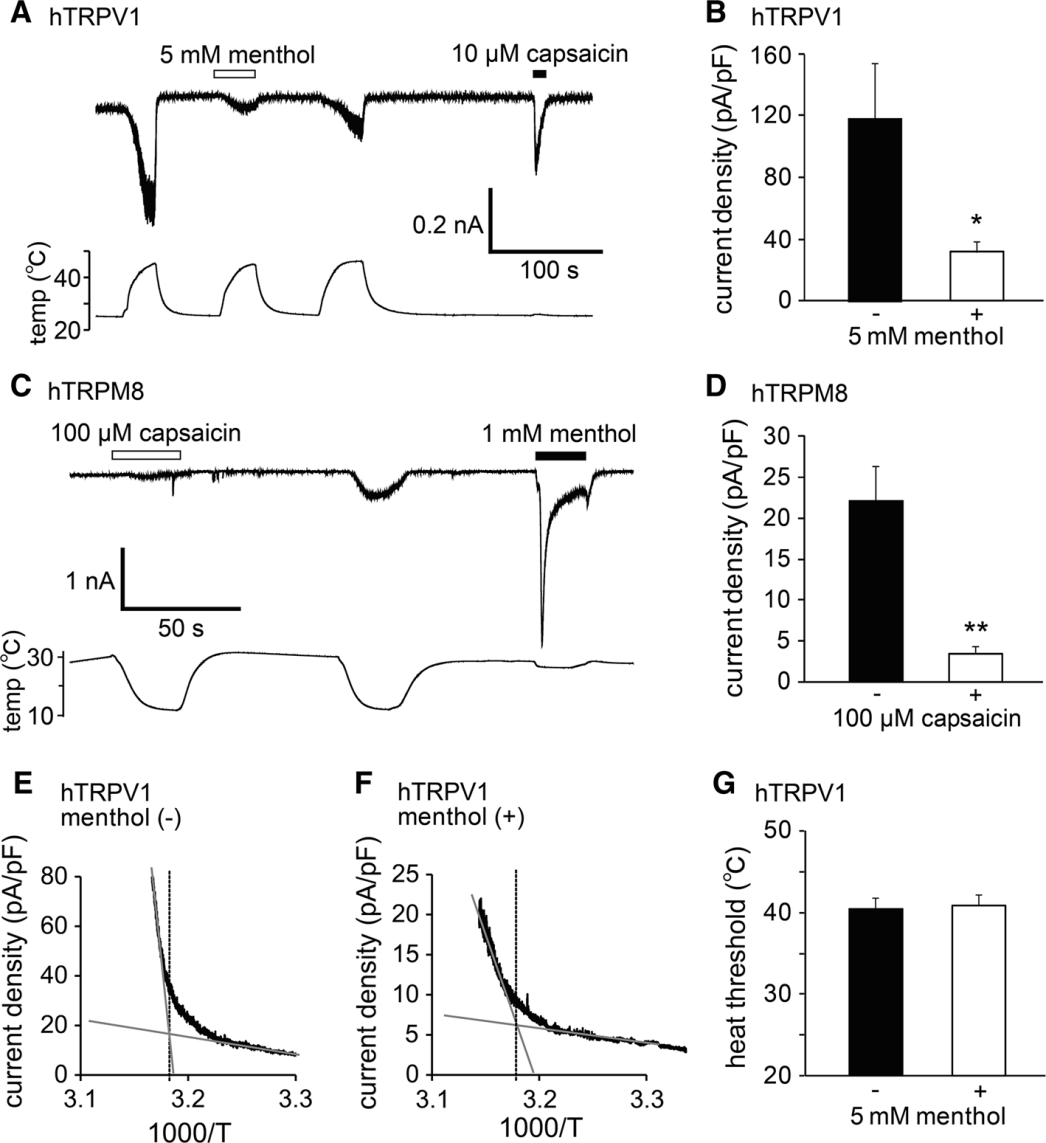
Changes in thermal sensitivity of hTRPV1 and hTRPM8 by menthol and capsaicin. **a** A representative trace of the hTRPV1-mediated heat-activated currents (*upper*) with changes in temperature (*lower*). The hTRPV1 response was confirmed by capsaicin (10 µM). **b** Comparison of the first heat-evoked hTRPV1-mediated current densities in the absence and presence of 5 mM menthol in HEK293T cells (*n* = 10–13). *p < 0.05. **c** A representative trace of the hTRPM8-mediated cold-activated currents (*upper*) with changes in temperature (*lower*). The hTRPM8 response was confirmed by menthol (1 mM). **d** Comparison of the first cold-evoked hTRPM8-mediated current densities in the absence and presence of 100 µM capsaicin in HEK293T cells (*n* = 10–12). **p < 0.01. **e**, **f** Temperature thresholds for heat-evoked hTRPV1 activation determined by Arrhenius plots from the data in the absence (42.3 °C; **e**) and presence (41.6 °C; **f**) of 5 mM menthol in HEK293T cells expressing hTRPV1. **g** The average temperature thresholds for hTRPV1 activation after the first heat stimulation in the absence and presence of 5 mM menthol in HEK293T cells (*n* = 6–7). Data are shown as the mean ± SEM

In order to examine more thoroughly the inhibitory effects of menthol and capsaicin on the thermal responses of hTRPV1 and hTRPM8, respectively, we performed patch-clamp experiments with HEK293T cells expressing hTRPV1 or hTRPM8. Menthol (5 mM) inhibited heat-evoked TRPV1-mediated currents in a reversible manner (Fig. 4a). The first heat-evoked currents in the presence of menthol (5 mM) were significantly smaller than those in the absence of menthol (36.8 ± 7.1 and 123.3 ± 35.5 pA/pF in the presence and absence of menthol, respectively; *p* < 0.05) (Fig. 4b). In a similar manner, capsaicin (100 µM) inhibited the cold-evoked TRPM8-mediated currents in a reversible manner (Fig. 4c), and the first cold-evoked currents in the presence of capsaicin (100 µM) were significantly smaller than those in the absence of capsaicin (3.4 ± 0.9 and 22.2 ± 3.4 pA/pF in the presence and absence of capsaicin, respectively; *p* < 0.01) (Fig. 4d). These results demonstrate that both the chemical and thermal responses of hTRPV1 and hTRPM8 were inhibited by menthol and capsaicin, respectively.

To determine whether the inhibition mediated by menthol and that mediated by capsaicin affected temperature thresholds, we then assessed changes in the temperature thresholds for hTRPV1 and hTRPM8 activation by constructing Arrhenius plots for hTRPV1-mediated and hTRPM8-mediated current responses. The temperature thresholds for hTRPV1 activation were not significantly different in the absence or presence of menthol (41.0 ± 1.3 vs. 41.2 ± 1.6 °C, respectively) (Fig. 4e–g). However, we were unable to generate Arrhenius plots for hTRPM8 currents because capsaicin completely inhibited cold-induced hTRPM8-mediated currents (Fig. 4c). These data indicate that menthol affected the size of the TRPV1 current without any changes the temperature thresholds for activation.

### The binding site of menthol in TRPV1 was distinguishable from that of capsaicin, whereas the binding site of capsaicin in TRPM8 modestly interacted with that of menthol

**Fig. 5:**
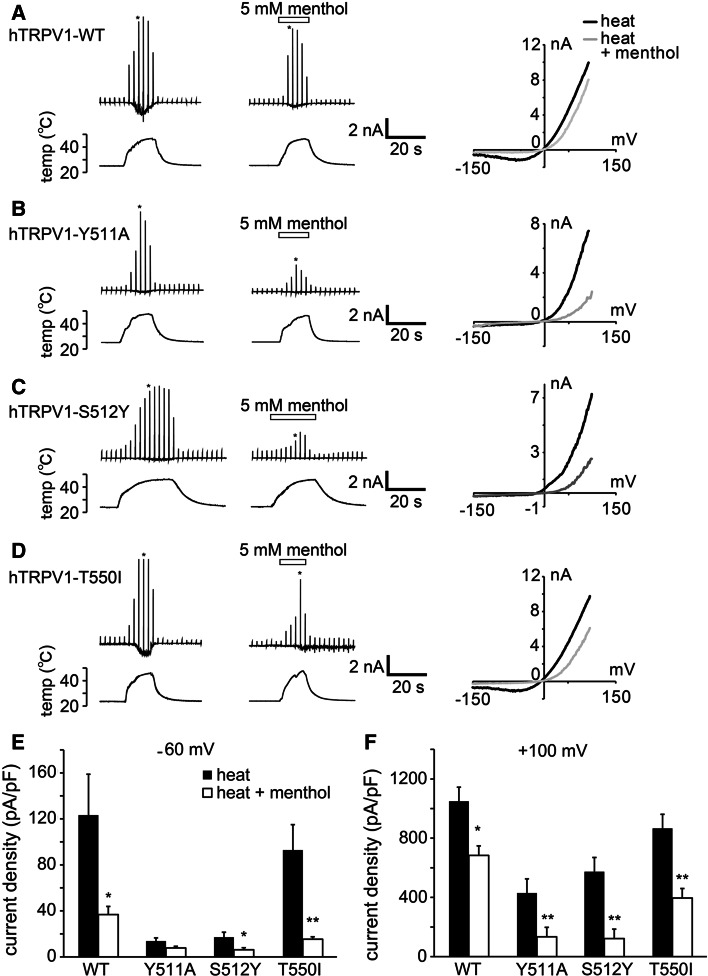
Comparison of the inhibitory effects of menthol on the currents of wild-type (*WT*) hTRPV1 and hTRPV1 mutants (*Y511A*,* S512Y*, T550I) expressed in HEK293T cells. **a–d** Representative traces of the first heat-activated currents (*upper*) in the absence and presence of menthol (5 mM) with temperature changes (*lower*) in HEK293T cells expressing WT (**a**), Y511A (**b**), S512Y (**c**), and T550I (**d**) hTRPV1. Current–voltage relationships (*right*) in the absence and presence of menthol at the points indicated by *asterisk* in the left traces. **e**, **f** Comparison of the current densities activated by the first heat stimulation in the absence or presence of menthol (5 mM) in HEK293T cells at −60 mV (**e**) and +100 mV (**f**). **p < 0.01, *p < 0.05. *n* = 6–13. Data are shown as the mean ± SEM

Menthol activated TRPM8 and inhibited TRPV1, whereas capsaicin activated TRPV1 and inhibited TRPM8. These results led us to hypothesize that the two compounds interact with similar sites on each channel in a competitive manner. To test this hypothesis, we examined whether menthol interacted with the same binding site as capsaicin on TRPV1 and whether capsaicin interacted with the same binding site as menthol on TRPM8. We also investigated whether menthol and capsaicin affected the heat-evoked hTRPV1 and cold-evoked TRPM8 activities, respectively, in mutant hTRPV1 and hTRPM8 channels in which capsaicin and menthol binding sites, respectively, had been mutated. Capsaicin has been reported to interact with Y511 and S512 in the cytosolic region linking transmembrane domains 2 and 3 of rat TRPV1 [44] and with T550 in the transmembrane domain 4 of *Oryctolagus cuniculus* [45], all of which are conserved in hTRPV1 (ESM Fig. 3a). Therefore, we examined the effects of menthol on the heat-evoked currents in the three hTRPV1 mutants (Y511A, S512Y and T550I) under naïve conditions. Because the heat-evoked inward currents of Y511A and S512Y mutants at −60 mV were very small (Fig. 5b, c, e), we examined the effects of menthol on the currents at both −60 and +100 mV. The effects of menthol on the heat-evoked currents appeared to be larger at −60 mV than at +100 mV in the wild-type (WT) and T550I channels (Fig. 5a, d–f). Although precise comparisons in the Y511A and S512Y channels at −60 mV were difficult due to the very small heat-evoked currents, menthol-induced inhibition of the mutant channels at +100 mV appeared to be large (Fig. 5b, c, e, f). Nonetheless, the levels of inhibition were generally similar between the WT and mutants, suggesting that a sharing of the three amino acids with capsaicin did not account for the inhibitory effects of menthol.

**Fig. 6:**
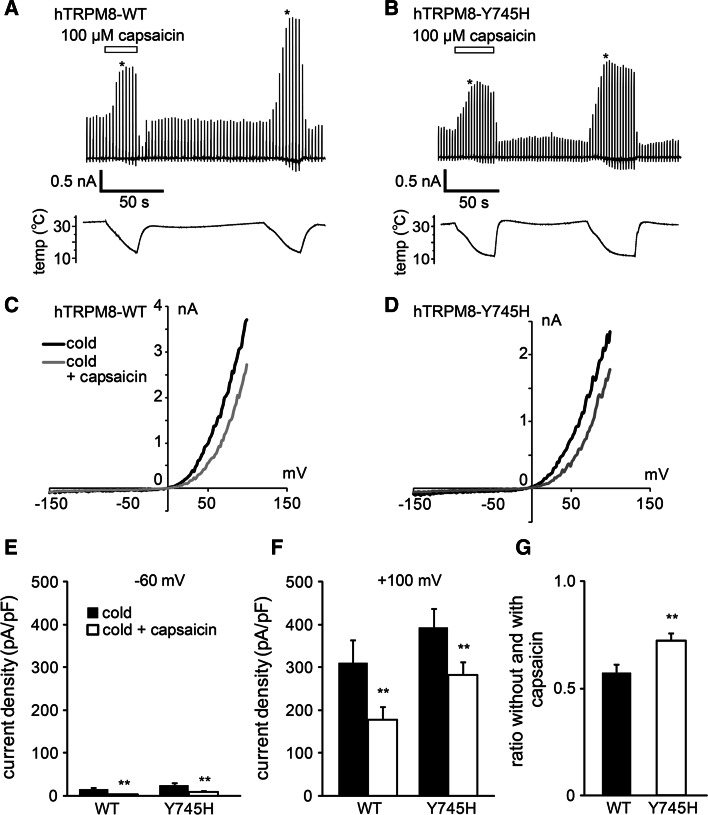
Comparison of the inhibitory effects of capsaicin on the currents of WT hTRPM8 and hTRPM8 mutant Y745H expressed in HEK293T cells. **a**, **b** Representative traces of the cold-activated currents (*upper*) in the absence and presence of capsaicin (100 μM) with temperature changes (*lower*) in HEK293T cells expressing WT (**a**) and Y745H (**b**). **c, d** Whole-cell current–voltage relationships of WT (**c**) and Y745H (**d**) expressed in HEK293T cells in the absence and presence of capsaicin at the points indicated by *asterisk* in the traces (**a**, **b**). **e**, **f** Comparison of the current densities activated by cold in the absence and presence of capsaicin (100 μM) at −60 mV (**e**) and +100 mV (**f**). **p < 0.01. *n* = 8–12. **g** Comparison of the ratios of the cold-activated currents at +100 mV without and with capsaicin from the data shown in **f**. **p < 0.01. Data are shown as the mean ± SEM

To the contrary, tyrosine 745 in the middle of transmembrane domain 2 has been identified as a crucial residue for the menthol sensitivity of mouse TRPM8 [48] that is conserved in hTRPM8 (ESM Fig. 3b). As already mentioned, cold-induced inward currents at −60 mV were very small, especially in the presence of capsaicin (Fig. 6a–e), thereby preventing us from comparing the effects at negative potentials. However, inhibition of cold-induced TRPM8-mediated currents by capsaicin appeared smaller at +100 mV in the Y745H mutant (Fig. 6d, f), and ratios without and with capsaicin were significantly larger in the Y745H mutant (*p* < 0.05) (Fig. 6g). Cold-induced TRPM8-mediated currents became small upon washout of capsaicin (Fig. 6a), which probably reflected the inhibition of basal TRPM8-mediated currents by residual capsaicin in the cells. We examined the involvement of two other amino acids (Y1005 and L1009) that have been reported to be involved in menthol action. However, the mutants (Y1005F and L1009R) did not provide currents of sufficient strength for analysis (data not shown). These findings suggest that Y745 was involved to some extent in capsaicin-induced hTRPM8 inhibition.

### Menthol inhibited sensory irritation caused by an hTRPV1 agonist in vivo

**Fig. 7:**
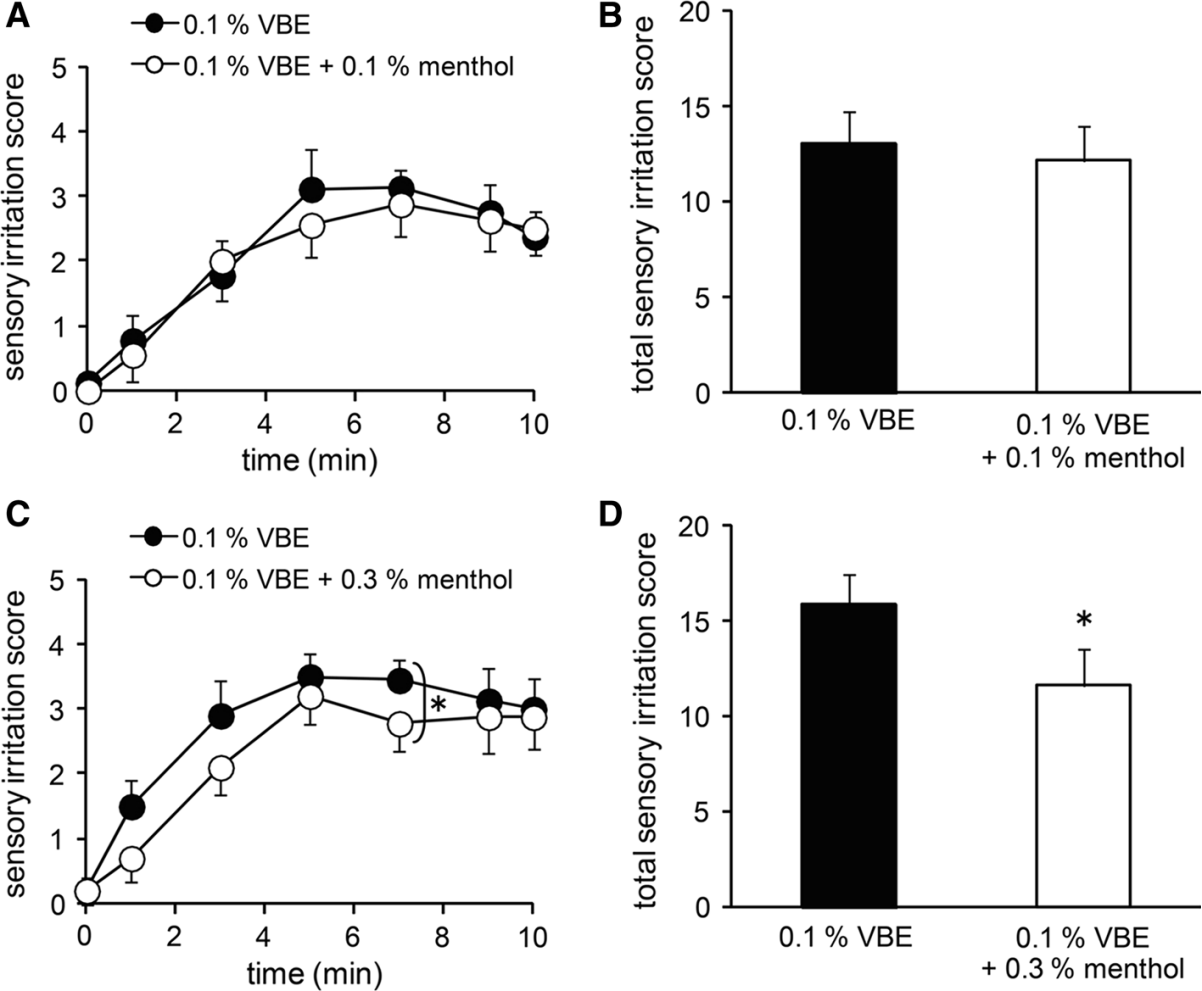
Inhibitory effects of menthol on the Vanillyl butyl ether (*VBE*)-induced sensory irritation in humans. **a**, **c** Changes in sensory irritation scores following the application of VBE (0.1 wt%) in the absence and presence of menthol (**a** 0.1 wt%; **c** 0.3 wt%) (*n* = 10 each). **b**, **d** Total scores of sensory irritation by VBE during 10 min in the absence and presence of menthol (**b** 0.1 wt%; **d** 0.3 wt%). *p < 0.05. Data are shown as the mean ± SEM

Menthol is frequently contained in topical analgesic drugs available in pharmacies, although its mechanism of action is still unknown. Based on the data shown above, we hypothesized that the analgesic effect of menthol may be through its inhibition of hTRPV1 activity. To confirm the possibility in vivo, sensitive human subjects were recruited for a sensory irritation test involving exposure to VBE, which is structurally similar to capsaicin [50]. We observed that VBE activated hTRPV1 (ESM Fig. 4a) and that this activation was inhibited by capsazepine (1 µM) (ESM Fig. 4b). VBE did not cause any current activation, but rather it decreased basal currents in the vector-transfected cells (ESM Fig. 4c). We confirmed that menthol (5 mM) inhibited the hTRPV1 activity evoked by VBE (100 µM) (ESM Fig. 4d, e), suggesting that tests involving both substances together might allow us to examine the inhibitory effect of menthol on sensory irritation by VBE. We found that VBE (0.1 wt%) caused sensory irritation and that this irritation gradually increased with increasing length of time after application (Fig. 7a, c). Although the VBE-induced sensory irritation scores were not reduced by concomitant application of 0.1 wt% menthol, they were significantly reduced by 0.3 wt% menthol (Fig. 7a–d). In human subjects, inhibition of VBE-induced irritation by menthol was smaller than the TRPV1 inhibitory effects observed in vitro. We also tested whether VBE might also activate hTRPA1 and found that VBE had a hTRPA1-activating ability, although the effects were observed only at 1 mM, whereas VBE activated hTRPV1 at lower concentrations (ESM Fig. 5).

## Discussion

In this study, we examined the effects of menthol on hTRPV1 and the effects of capsaicin on hTRPM8. The hTRPV1 currents induced by capsaicin were inhibited by menthol in a dose-dependent manner, and the hTRPM8 currents induced by menthol were inhibited by capsaicin in a dose-dependent manner. Our findings suggest that agonists of TRPV1 and TRPM8 exhibit mutually inhibitory effects on these channels.

In the human study, we examined whether menthol could inhibit sensory irritation caused by VBE. In the clinical setting, especially in the cosmetic research field, it is well-known that VBE causes skin irritation. Neither capsaicin nor resiniferatoxin is used for human skin studies. Surprisingly, we found that VBE activated both hTRPV1 and hTRPA1, which possibly explains the marked irritation produced by VBE when applied to humans. Our in vitro study showed that the IC_50_ value for menthol-induced hTRPV1 inhibition was 1.2 ± 0.2 mM and that it appeared that a very high level of menthol, 10 mM, completely inhibited hTRPV1 currents (Fig. 2). However, 0.3 wt% menthol, which exhibited anti-nociceptive effects in the in vivo study (Fig. 7), corresponded to 19.2 mM, which is within the attainable concentration range in clinical use [37, 38]. Menthol is reported to cause analgesic effects through several different mechanisms, including those which are TRPM8-dependent and -independent [34–39]. Menthol also activates hTRPA1 at higher concentrations than those which activate hTRPM8 [51]. In our human study, we observed the net result of hTRPV1 activation resulting from VBE, inhibition of VBE-evoked hTRPV1 activation by menthol, menthol-evoked hTRPA1 activation, VBE-evoked hTRPA1 activation, and other mechanisms. This complicated network of mechanisms could partly explain the small inhibition of VBE-induced irritation by menthol in human subjects. Nevertheless, the observation that high concentrations of menthol (0.3 %) (which could activate hTRPA1, leading to greater irritation) inhibited VBE-induced irritation could be significant. Thus, the reduction of VBE-induced irritation by 0.3 wt% menthol in vivo may be partly attributable to the inhibition of hTRPV1 activity by menthol shown in the in vitro study.

Preparations containing menthol are used topically to relieve neuralgia in traditional Chinese and European medicine [33]. In addition, mint oil has been reported to alleviate thermally elicited pain and post-herpetic neuralgia, and orally applied menthol can achieve short-term analgesia [52]. Furthermore, in mice, oral or intracerebroventricular application of menthol was observed to decrease nociceptive responses in the hot-plate test and acetic acid writhing test [53]. Despite the reports on the analgesic actions of menthol in the literature, the mechanism of action has not been fully clarified, although reduced menthol-induced anti-nociception was reported in TRPM8-deficient mice [39]. Inhibition of TRPV1 by menthol, as shown in the current study, could be one of the underlying mechanisms accounting for the analgesic effects of menthol observed in rodents and humans. However, menthol could also exhibit its anti-nociceptive effects through activation of TRPM8 and other mechanisms, as reported earlier [37, 38]. TRPV1 is activated by capsaicin as well as by heat, protons, and some endogenous substances known to be associated with tissue inflammation [4–6]. Since TRPV1 acts as an integrator of painful stimuli, TRPV1 antagonists can be viewed as promising and novel types of analgesics [40–43]. A number of potent, small TRPV1 antagonists, such as capsazepine, BCTC, CTPC, AMG9810, and SB-452533 [54–58], have advanced into clinical trials for the evaluation of their analgesic activity. Although some of these antagonists reduced noxious heat sensation, hyperthermia as a serious side effect often led to their withdrawal from the clinical trials. Thus, novel approaches to the development of anti-TRPV1 antagonists are needed, and this study shows that derivatives of menthol could be promising molecules to develop TRPV1 antagonists.

Agonists of TRPV1 and TRPM8 seem to exhibit mutual interactions for these channels. What is the physiological significance of the apparent reciprocal interaction? TRPM8 is not generally co-expressed with TRPV1 in primary afferent neurons [20–23], suggesting that the information conducted by TRPM8-expressing neurons and TRPV1-expressing neurons could influence each other. The data presented here suggest the possibility that menthol-induced TRPM8-mediated cold sensation could be enhanced by the inhibition of TRPV1 and that capsaicin-induced TRPV1-mediated heat sensation could be enhanced by the inhibition of TRPM8. The enhancement could work to strengthen the difference between TRPV1 and TRPM8 activities in some specific concentration ranges. Although we currently do not know the physiological significance of TRPM8 inhibition by capsaicin, the reciprocal interaction could lead to the enhancement of efficacious TRPV1-mediated nociceptive signals.

## Electronic supplementary material

Supplementary material 1 (DOCX 225 kb)

Supplementary material 2 (JPEG 320 kb)

Supplementary material 3 (JPEG 170 kb)

Supplementary material 4 (JPEG 326 kb)

Supplementary material 5 (JPEG 516 kb)

Supplementary material 6 (JPEG 179 kb)

## Abbreviations

HEK293T: Human embryonic kidney-derived 293T
TRPM8: Transient receptor potential melastatin 8
TRPV1: Transient receptor potential vanilloid 1
VBE: Vanillyl butyl ether
