# Supplementary material for: Reciprocal effects of capsaicin and menthol on thermosensation through regulated activities of TRPV1 and TRPM8
Source: J Physiol Sci. 2015 Dec 8;66(2):143–55. doi: 10.1007/s12576-015-0427-y (PMC4752590; doi:10.1007/s12576-015-0427-y)
Supplement: Supplementary file 1 — Supplementary material 1 (DOCX 225 kb) [file 12576_2015_427_MOESM1_ESM.docx]

**Supplementary Figure 1.** **Effects of menthol and capsaicin on HEK293T cells expressing hTRPV1, TRPM8 or vector alone.** (A, B) Representative traces of the whole-cell currents in the presence of menthol (10 mM) in a vector-transfected HEK293T cell (A) or a hTRPV1-expressing cell (B) in the presence of extracellular Ca^2+^. (C) A representative trace of the whole-cell currents in the presence of capsaicin (1 mM) in a hTRPM8-expressing cell in the presence of extracellular Ca^2+^.

**Supplementary Figure 2. Inhibitory effects of menthol on hTRPV1 current induced by various concentrations of capsaicin.** Comparison of the current densities activated by 0.01, 0.03 or 0.1 μM capsaicin in the absence and presence of menthol (1 mM). n = 5-8.

**Supplementary Figure 3. Schematic figures representing the topological structures of hTRPV1 (A) and hTRPM8 (B).** Barrels represent the putative transmembrane regions, and the circle indicates the proposed location of the tyrosine residue at position 511, the serine residue at position 512 and the threonine residue at position 550 on hTRPV1 and the tyrosine residue at position 745 on hTRPM8.

**Supplementary Figure 4.** **Effects of VBE on HEK293T cells expressing hTRPV1 or vector alone.**  (A - D) Representative traces of the whole-cell currents in the presence of VBE (1 mM) in hTRPV1-expressing HEK293T cells (A, B, D) or a vector-transfected cell (C) in the presence of extracellular Ca^2+^. VBE (100 µM)-evoked hTRPV1 currents that were inhibited by capsazepine (1 μM, B) or menthol (5 mM, D) in the presence of extracellular Ca^2+^. (E) Current-voltage relationships in the absence or presence of menthol at the points indicated by * in the left trace (D).

**Supplementary Figure 5.** **Effects of VBE on HEK293T cells expressing hTRPA1.**  (A) A representative trace of the whole-cell current in the presence of VBE (1 mM) in a hTRPA1-expressing HEK293T cell in the presence of extracellular Ca^2+^. (B) Comparison of the current densities activated by 0.1 and 1 mM VBE on hTRPV1 and hTRPA1. n = 5-6.
