# Supplementary figures and images for: Reciprocal effects of capsaicin and menthol on thermosensation through regulated activities of TRPV1 and TRPM8
Source: J Physiol Sci. 2015 Dec 8;66(2):143–55. doi: 10.1007/s12576-015-0427-y (PMC4752590; doi:10.1007/s12576-015-0427-y)

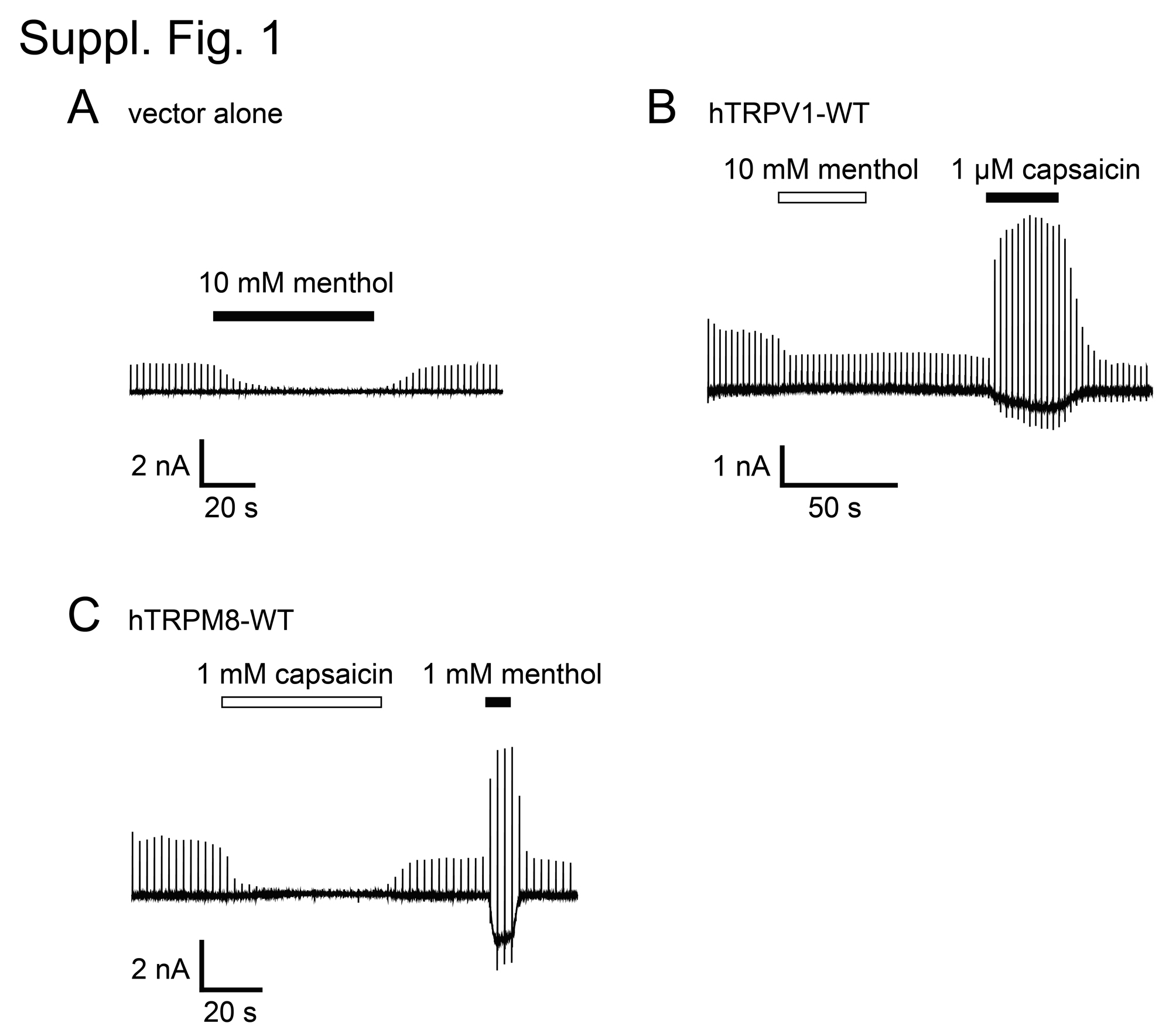

Supplement: Supplementary file 2 — Supplementary material 2 (JPEG 320 kb) [file 12576_2015_427_MOESM2_ESM.jpg]

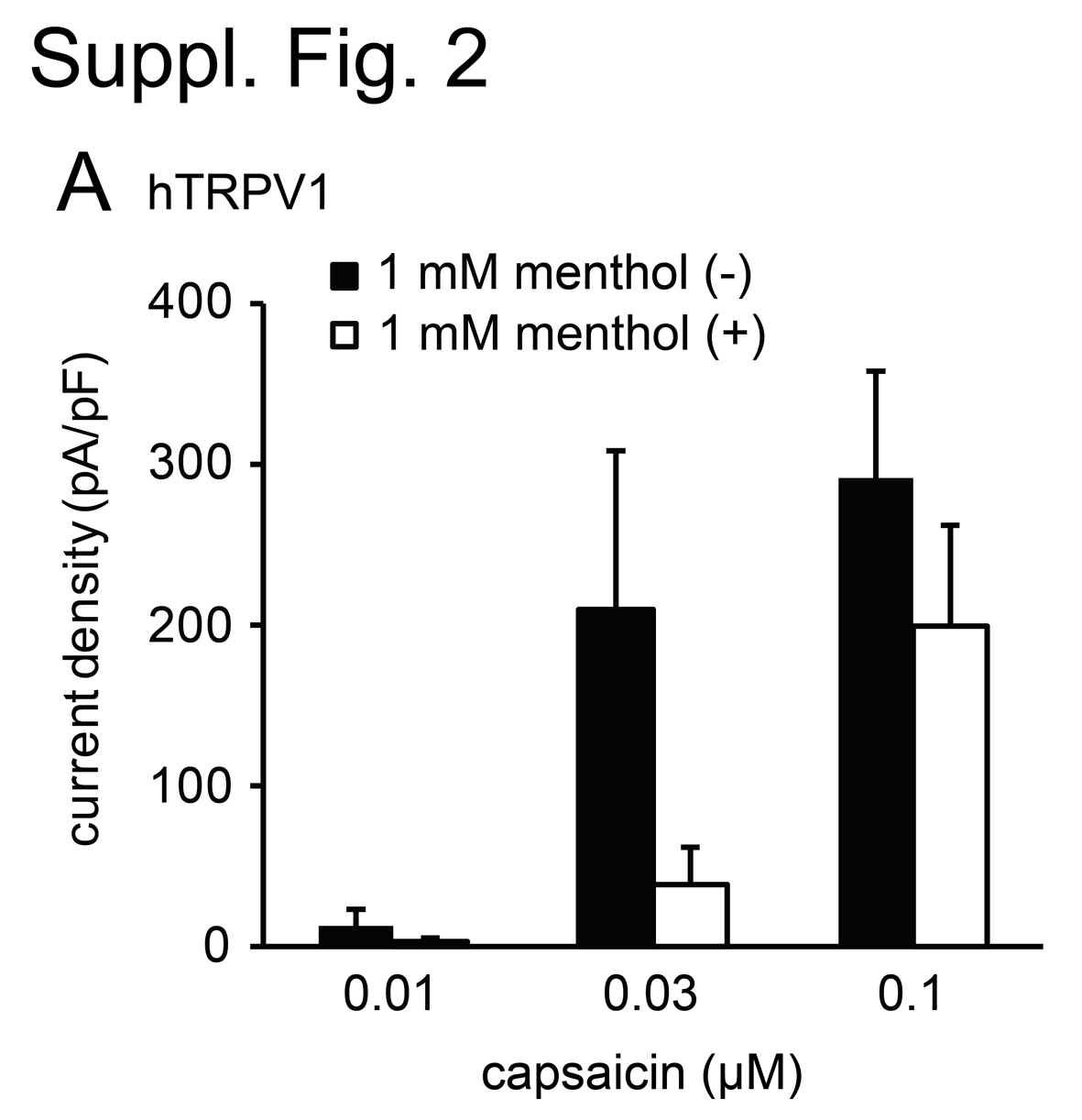

Supplement: Supplementary file 3 — Supplementary material 3 (JPEG 170 kb) [file 12576_2015_427_MOESM3_ESM.jpg]

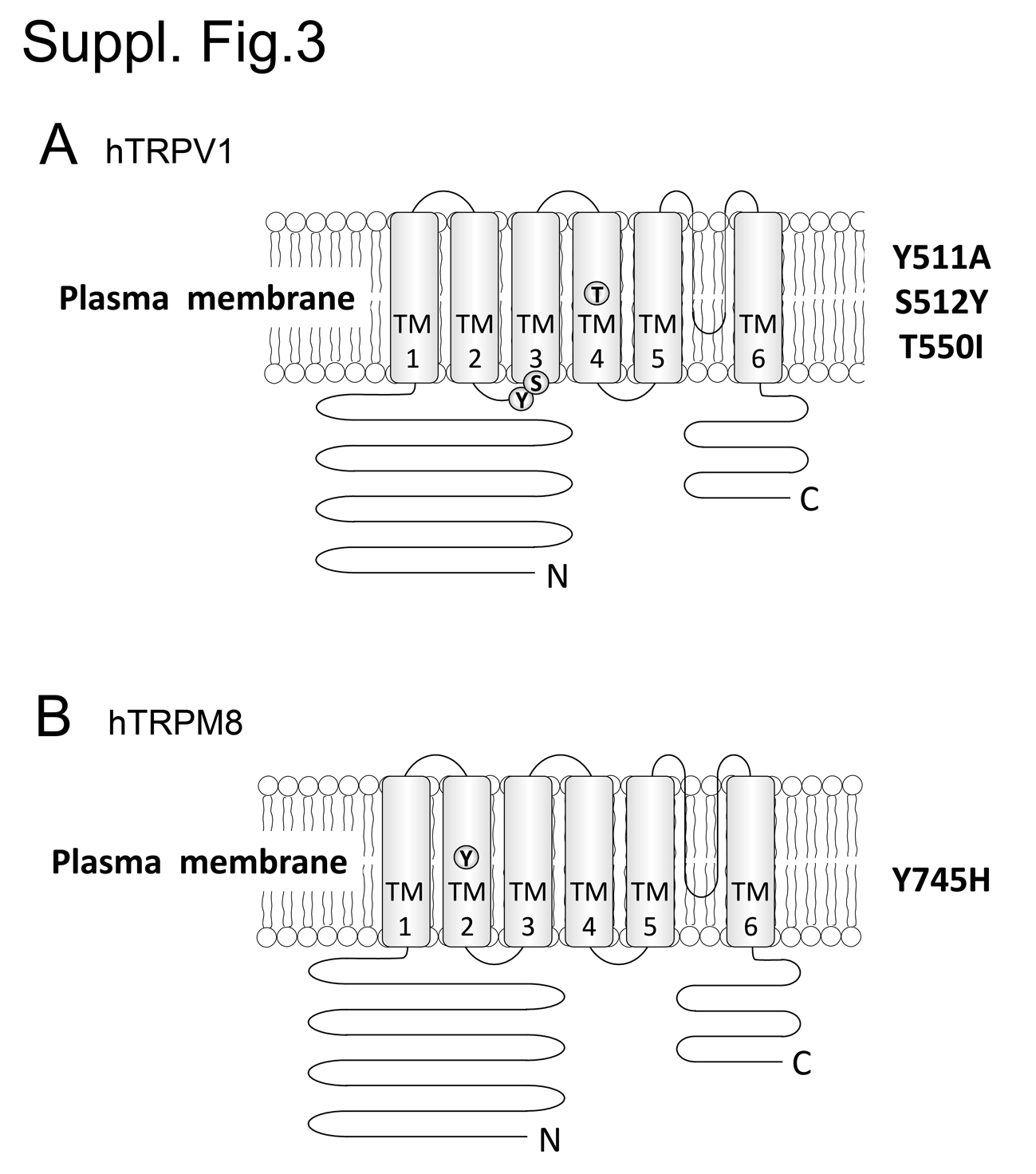

Supplement: Supplementary file 4 — Supplementary material 4 (JPEG 326 kb) [file 12576_2015_427_MOESM4_ESM.jpg]

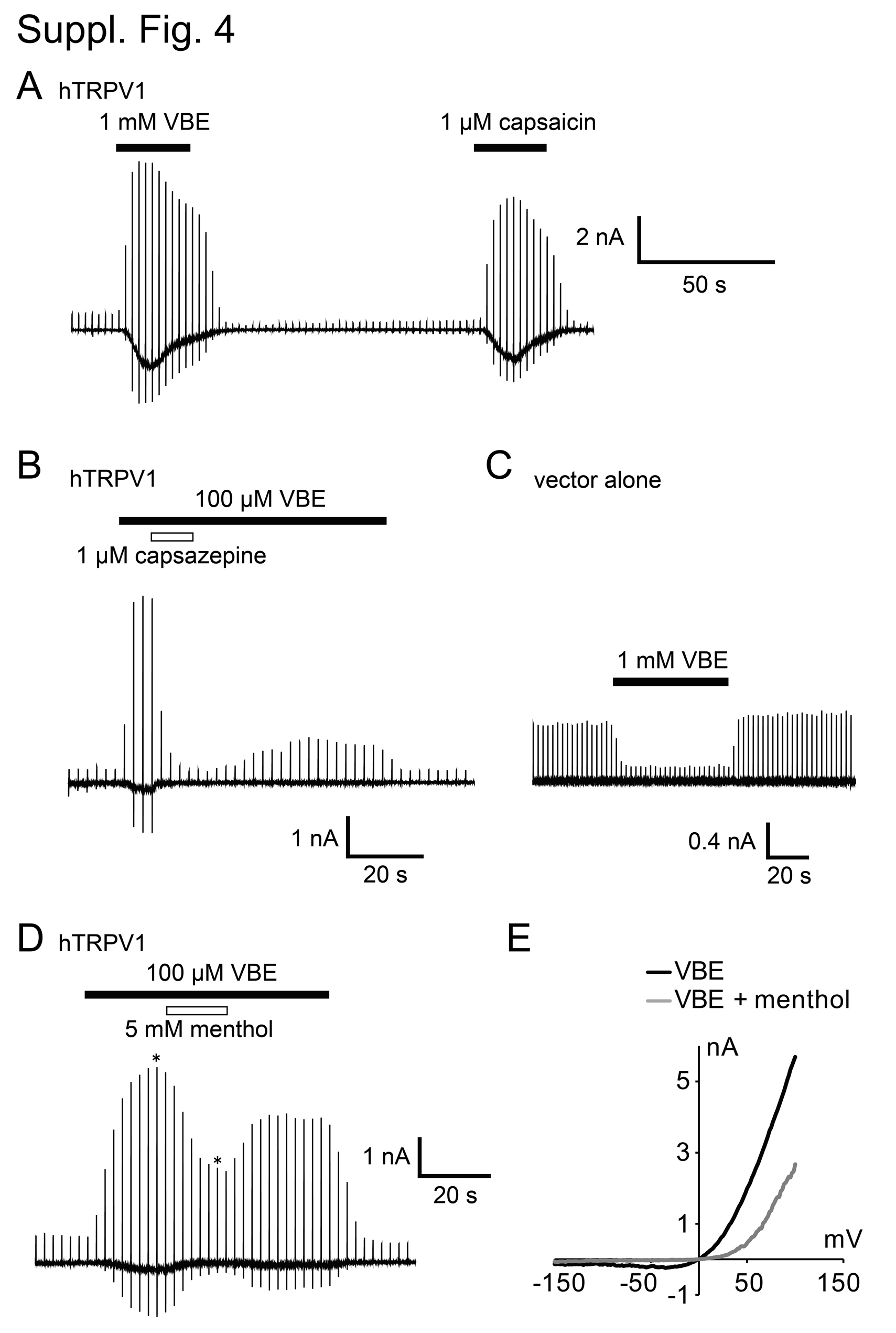

Supplement: Supplementary file 5 — Supplementary material 5 (JPEG 516 kb) [file 12576_2015_427_MOESM5_ESM.jpg]

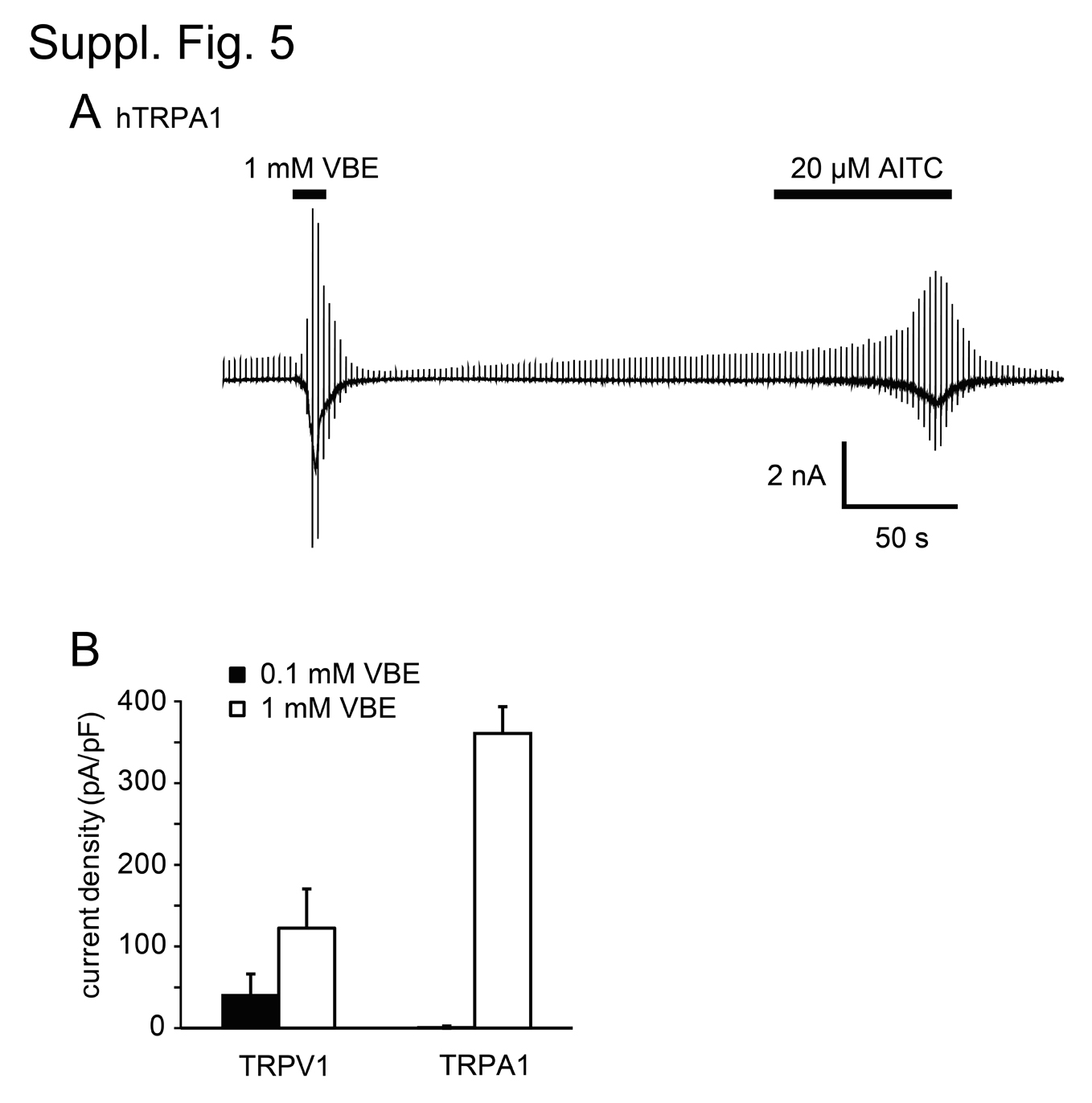

Supplement: Supplementary file 6 — Supplementary material 6 (JPEG 179 kb) [file 12576_2015_427_MOESM6_ESM.jpg]
